# Supplementary figures and images for: Changes in Alcohol Consumption during the COVID-19 Pandemic: Evidence from Wisconsin
Source: Int J Environ Res Public Health. 2023 Mar 29;20(7):5301. doi: 10.3390/ijerph20075301 (PMC10094098; doi:10.3390/ijerph20075301)

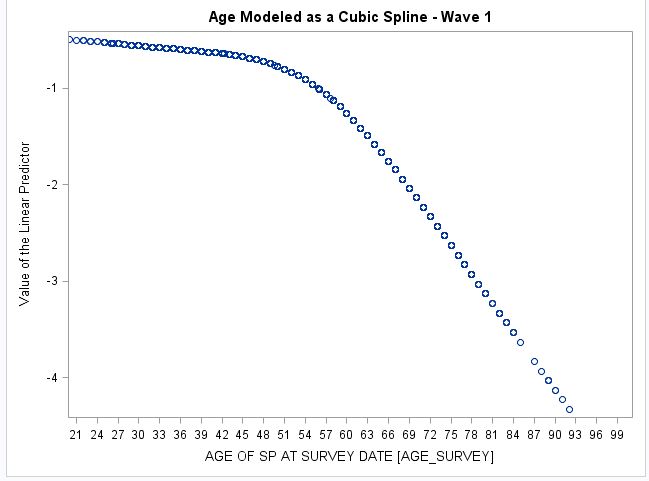

Supplement: Supplementary file 1 [file ijerph-20-05301-s001.zip › Supplementary Materials/FigureS1.JPG]

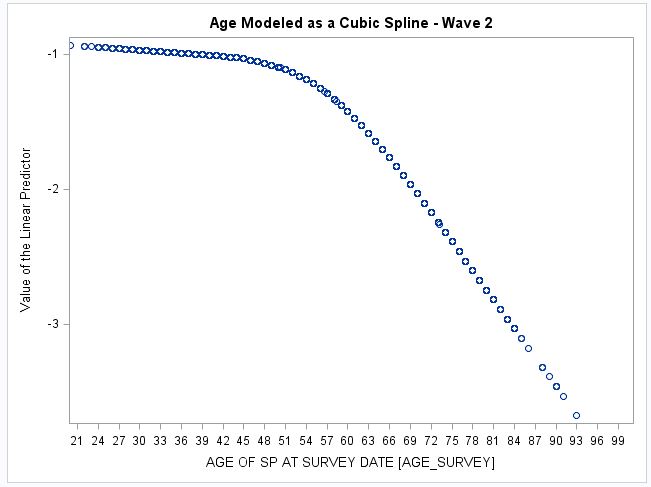

Supplement: Supplementary file 1 [file ijerph-20-05301-s001.zip › Supplementary Materials/FigureS2.JPG]

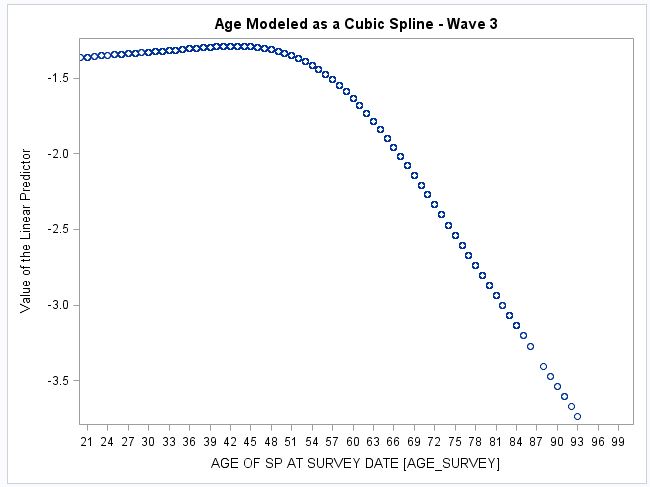

Supplement: Supplementary file 1 [file ijerph-20-05301-s001.zip › Supplementary Materials/FigureS3.JPG]
